# Bacteria Modify Their Sensitivity to Chemerin-Derived Peptides by Hindering Peptide Association With the Cell Surface and Peptide Oxidation

**DOI:** 10.3389/fmicb.2020.01819

**Published:** 2020-08-11

**Authors:** Urszula Godlewska, Bernadetta Bilska, Paweł Majewski, Elzbieta Pyza, Brian A. Zabel, Joanna Cichy

**Affiliations:** ^1^Department of Immunology, Faculty of Biochemistry, Biophysics and Biotechnology, Jagiellonian University, Kraków, Poland; ^2^Department of Cell Biology and Imaging, Institute of Zoology and Biomedical Research, Jagiellonian University, Kraków, Poland; ^3^Palo Alto Veterans Institute for Research, VA Palo Alto Health Care System, Palo Alto, CA, United States

**Keywords:** chemerin, antimicrobial peptide, skin, psoriasis, sigma factor

## Abstract

Chronic inflammatory skin diseases like psoriasis alter the local skin microbiome and lead to complications such as persistent infection with opportunistic/pathogenic bacteria. Disease-associated changes in microbiota may be due to downregulation of epidermal antimicrobial proteins/peptides, such as antimicrobial protein chemerin. Here, we show that chemerin and its bioactive derivatives have differential effects on the viability of different genera of cutaneous bacteria. The lethal effects of chemerin are enhanced by bacterial-derived ROS-induced chemerin peptide oxidation and suppressed by stationary phase sigma factor RpoS. Insight into the mechanisms underlying changes in the composition of cutaneous bacteria during autoreactive skin disease may provide novel ways to mobilize chemerin and its peptide derivatives for maximum antimicrobial efficacy.

## Introduction

To prevent pathologic outcomes, the skin must continuously confront a wide and diverse array of bacterial challenges. The primary line of defense relies on keratinocytes and their ability to secrete antimicrobial peptides. Among keratinocyte-derived factors equipped with antimicrobial potential is chemerin. Chemerin is a secreted multifunctional protein that is known mainly for its properties to support immune cell infiltration to inflammatory sites and regulate differentiation of adipocytes (Wittamer et al., 2003; Zabel et al., 2005b; Goralski et al., 2007). Chemerin is secreted as a functionally inert precursor protein (Chem163S, with number and capital letter referring to the terminal amino acid position and single amino acid code). Chem163S can be converted to active isoforms through posttranslational carboxyl-terminal processing. Proteolytic cleavage at serine 157 in the carboxyl-terminus of Chem163S results in generation of Chem157S isoform that is effective in triggering chemotaxis of several types of immune cells (Yamaguchi et al., 2011). This isoform also exhibits much stronger growth inhibitory potential compared with Chem163S against bacteria (Kulig et al., 2011; Godlewska et al., 2017). However, in contrast to C-terminal region responsible for chemotactic potential, antimicrobial activity is mainly associated with a domain localized in the middle of the chemerin sequence, Val^66^-Pro^85^ peptide (p4), which embodies the majority of chemerin’s anti-microbial activity (Banas et al., 2013). Therefore, to exhibit potent antimicrobial activity, holoprotein Chem163S requires removal of a terminal inhibitory peptide, possibly to enable structural accessibility of its antimicrobial domain, and/or a release of its central antimicrobial peptide. Chem157S has been isolated from human biological specimens, including ascites and serum (Zabel et al., 2006). Although cutaneous chemerin isoforms remain to be identified, endogenous chemerin is largely responsible for the natural antimicrobial activity present in keratinocyte secretions (Banas et al., 2013), suggesting that chemerin isoform(s) capable of controlling bacteria growth are generated in the epidermis. Given that several chemerin receptors can retain chemerin on the cell surface (Zabel et al., 2014), structural features/changes in chemerin structure following binding to these receptors might enable the p4 domain to interact with bacteria. Since chemerin is highly susceptible to proteolytic cleavage (Zabel et al., 2014), it is also likely that p4 can be released from chemerin by endogenous (Wittamer et al., 2005; Zabel et al., 2005a; Schultz et al., 2013) or bacteria-derived proteases (Kulig et al., 2007) and act independently of the rest of chemerin protein. Synthetic p4 is effective in treating experimental *S. aureus* skin infections (Godlewska et al., 2019), suggesting that it could impact the clinical management of *S. aureus* and potentially other bacteria-mediated skin pathologies.

Despite our growing understanding of the local and systemic role of chemerin in immunity, the function of this protein at body barriers remains poorly understood. Chemerin is abundantly expressed by keratinocytes in healthy skin but it is markedly downregulated in the epidermis of patients suffering from the autoinflammatory skin disease psoriasis (Albanesi et al., 2009). Since the overall microbial community (microbiota) of normal and psoriatic skin can differ substantially (Gao et al., 2008; Chang et al., 2018), active chemerin derivatives may contribute to skin pathophysiology by shaping the composition of the skin microbiota.

Here, we demonstrate differentiating potentials of chemerin isoforms and p4 in controlling cutaneous bacteria and identify novel bacteria-mediated mechanisms that influence the antimicrobial activity of chemerin peptide, which may modify the genus-, species-, and strain-level structure of the skin microbiome.

## Materials and Methods

### Bacterial Strains

The following laboratory or clinical reference strains were used in the study: *Escherichia coli* HB101, *Escherichia coli* DH5α, and *Escherichia coli* NiCo21(DE3)*; Staphylococcus aureus* strain 8325-4, *Staphylococcus epidermidis* DSM 20044, *Staphylococcus hominis* DSM 20328, *Staphylococcus capitis* DSM 20326, *Staphylococcus caprae* DSM 20608, *Streptococcus mitis* DSM 12643, *Corynebacterium simulans* DSM 44392, *Corynebacterium tuberculostearicum* DSM 44922, *Cutibacterium acnes* DSM 16379. The clinical reference strains were obtained from DSMZ German Collection of Microorganisms and Cell Cultures GmbH (Braunschweig). *Rhodobacter capsulatus* (*R. capsulatus*) strains included *R. capsulatus* pMTS1/MTR*bc*1 strain overproducing cytochrome *bc*_1_ (WT), the MT-RBC1 knockout strain with a deletion of the operon coding for cytochrome *bc*_1_, G167P strain with glycine 167 to proline mutation in the cytochrome *b* protein of the *bc*_1_complex, and 2Ala with alanine insertion mutation in the iron-sulfur protein subunit of the *bc*_1_complex (Borek et al., 2008, 2015). *R. capsulatus* strains were kindly donated by Dr. A. Osyczka (Dept. of Molecular Biophysics, Jagiellonian University, Kraków, Poland).

### Production of Recombinant Human Chemerin Isoforms

Recombinant human full-length chemerin variant Chem163S and chemerin variant Chem157S, lacking 6 aa at C-terminus, were produced in *Escherichia coli*. DNA fragments corresponding to the desired chemerin proteins were amplified by PCR and cloned into the pNIC28-Bsa4 expression vector (Addgene, LGC Standards, Teddington, United Kingdom) at a site preceded by the sequence coding for hexahistidine tag, using the overlap-extension PCR (Bryksin and Matsumura, 2010). Both constructs lacked the first 20 aa native chemerin signal peptide. The identity of the created pNIC28-Bsa4-chem157 or pNIC28-Bsa4-chem163 constructs was verified by sequencing (Genomed, Warsaw, Poland). Recombinant chemerin isoforms were expressed and purified in *E. coli* strain NiCo21(DE3) (New England Biolabs, MA, United States), transformed with the plasmids described above. Recombinant proteins were purified from the inclusion bodies of bacteria transformants, using Ni-Sepharose 6 Fast Flow, followed by Q Sepharose Fast Flow (both from GE Healthcare, Uppsala, Sweden). The recombinant chemerin variants were first eluted with 500 mM imidazole, and in the second purification step with 25–35 mM (Chem163S) or 35–50 mM (Chem157S) NaCl and dialyzed against PBS. The obtained proteins were routinely >90% pure as assessed by SDS-PAGE and Coomassie Blue staining.

### Chemerin Peptide 4 (p4)

Peptide p4 was chemically synthesized by ChinaPeptide (Shanghai, China) at ≥95% purity. Biotin- or FITC-labeled p4 were synthesized by CASLO (Kongens Lyngby, Denmark) at ≥95% or ≥98% purity. Biotin was added directly at the N-terminus of p4. For FITC-labeled p4, C-terminal lysine was added to p4 and FITC was conjugated to the side chain of this C-terminal lysine. Both biotin-labeled and FITC-labeled p4 displayed similar antimicrobial activity to unmodified p4.

### Antimicrobial Microdilution Assay (MDA)

For antimicrobial experiments all staphylococci and *C. acnes* were cultured in brain heart infusion broth (BHI) (Sigma-Aldrich). *S. mitis* and *C. simulans* were cultured in BHI supplemented with 0.3% yeast extract (Sigma-Aldrich), whereas *C. tuberculostearicum* was grown in BHI supplemented with 0.3% yeast extract and 1% Tween 80 (Sigma-Aldrich). Bacteria were grown under aerobic condition with two exceptions. *C. acnes* was grown in BHI in an anaerobic atmosphere using a GasPak^TM^ EZ Anaerobe Pouch System (BD), and *S. mitis* was grown in a 5% CO2 atmosphere using a GasPak^TM^ EZ CO2 Pouch System (BD). To determine the antimicrobial activity of chemerin and peptide p4 against skin-associated species, bacteria in mid-logarithmic phase were diluted to 4 × 10^5^ CFU/ml with PBS containing a series of 2-fold dilution of p4, chemerin isoforms Chem157S and Chem163S (5 μM), or PBS (control) and incubated for 2 h. The number of viable bacteria were enumerated by colony forming units (CFU) counting. In the experiments with *R. capsulatus* strains, bacteria were grown protected from light in mineral-peptone-yeast extract (MPYE) at 30°C. Bacteria in mid-logarithmic phase were diluted to 4 × 10^5^ CFU/ml with PBS containing 1% (v/v) of medium and preincubated with 2 μM antimycin (Sigma-Aldrich) for 15 min to promote ROS production by *bc*_1_, before adding 2.5 μM p4. The number of viable bacteria were enumerated by CFU counting. To determine whether susceptibility to p4 is growth-dependent, *E. coli* WT and *rpoS* mutants were cultured in Luria-Bertani (LB) medium (Sigma-Aldrich), and when required supplemented with antibiotics. Bacteria in logarithmic or stationary growth phase were diluted to 4 × 10^5^ CFU/ml with PBS containing a series of 2-fold dilution of p4 or PBS (control) and incubated for 2 h. The surviving bacteria were enumerated by CFU counting.

### ATP Determination

Total ATP levels were determined using The BacTiter-Glo viability assay kit^®^ (Promega), following the manufacturer’s instructions. In brief, bacteria in mid-logarithmic phase were diluted to 8 × 10^6^ CFU/ml with PBS containing a series of 2-fold dilution of p4 or PBS (control). After incubation with p4, BacTiter-Glo reagent was added to each well (1:1 [v:v]) and incubated in darkness for 5 min at room temperature (RT). The luminescent signal was recorded using Tecan Infinite M200 Plate Reader.

### Fluorometric Measurement of Membrane Potential

Membrane potential was measured by using a voltage-sensitive dye DiSC3(5) (Sigma-Aldrich). *E. coli* or *S. epidermidis* cultures were grown in BHI to the mid-logarithmic phase and diluted to an OD600 of 0.2. 15 mM. EDTA was added to *E. coli* dilution to facilitate the uptake of the DiSC3(5). DiSC3(5) was added to each well to a final concentration of 1 μM followed by 25 μM p4 to monitor the dissipation of membrane potential. Gramicidin (1 μM) (Sigma-Aldrich) was used as a positive control. The fluorescence at an excitation wavelength of 615 nm and an emission wavelength of 665 nm was measured using Tecan Infinite M200 Plate Reader.

### Fluorescence Microscopy

*Escherichia coli* in logarithmic or stationary phase (1 × 10^8^ CFU) were incubated for indicated time with FITC-labeled p4. Cells were washed three times with PBS to remove the peptide, attached to slides by cytospin centrifugation, fixed in 3.7% paraformaldehyde (Sigma-Aldrich), and counterstained with 1 μg/mL Hoechst dye 33258 (Life Technologies) for 30 min at RT. Images were captured with a fluorescence microscope (Eclipse; Nikon) and analyzed using NIS-Elements (Nikon) Imaging Software AR 5.01.00. Nucleoid lengths were measured automatically. Stained nucleoids less than 42 μm in length or obtained from cell duplicates were excluded from data analysis. Quantification of FITC-p4 labeled cells was done manually using NIS-Elements software.

### DNA Binding Assay

Total genomic DNA from *E. coli* was isolated using a DNA extraction kit (Thermo Fisher Scientific), according to the manufacturer’s instruction. Gel retardation experiments were performed by mixing 150 ng of the DNA and increasing amounts of p4 in buffer containing 10 mM Tris–HCl, 1 mM EDTA, pH 8.0. The mixtures were incubated at RT for 30 min, and subsequently analyzed by electrophoresis on a 0.8% agarose gel in the TBE buffer (90 mM Tris-borate, 2 mM EDTA, pH 8.3).

### qRT-PCR

The total fraction of RNAs was isolated from the *E*. *coli* HB101 using Total RNA Zol-Out^TM^ (A&A Biotechnology) and converted to cDNA using NxGen M-MulV reverse transcriptase (Lucigen) with random hexamers (Promega). Real time PCR was performed on the C1000 Thermal cycler (CFX96 Real Time System, Bio-Rad) using SYBR Green I containing universal PCR master mix (A&A Biotechnology) and primers specific for *16S rRNA*, 5′- TGTSTGCAYGGYTGTCGTCA-3′, 5′- ACGTCRTCCMCACCTTCCTC-3′; *gyrA*, 5′- CGAGCG CGGATATACACCTT-3′, 5′- TCCGGTATCGCCGTAGGTAT-3′ (Genomed).

### Transmission Electron Microscopy (TEM)

5 × 10^8^ *E. coli, S. epidermidis*, or *S. aureus* cells were treated with p4 or vehicle (PBS) for 2 h at 37°C. *E. coli* cell pellets were fixed in 2% glutaraldehyde in 0.1 M sodium cacodylate buffer (pH 7.4) overnight at 4°C while Staphylococci pellets were washed three times in PBS for 5 min and fixed overnight in 2.5% glutaraldehyde in PBS at 4°C. *E. coli* was then washed in 0.1M sodium cacodylate buffer, post-fixed in 1% osmium tetroxide in 0.1M cacodylic buffer for 1 h at RT, washed again two times in the buffer and distilled water, and stained “en bloc” with 2% uranyl acetate aqueous solution for 1 h at RT. *S. aureus* was washed in PBS and post-fixed with 1% osmium tetroxide for 2 h at 4°C. Samples were embedded in epoxy resin (PolyBed 812; Polyscienses, Inc., Warrington, PA, United States) after dehydration in graded ethanol series (50–100%) and in propylene oxide. Ultrathin sections (65 nm) were cut using ultramicrotome (Leica EM UC7) and post-stained with uranyl acetate and lead citrate. Specimens were observed using a transmission electron microscope (JEOL JEM2100) operating at an accelerating voltage of 80 kV.

### Immunogold Labeling

*Escherichia coli* bacteria were treated with p4-biotin or unlabeled p4 as a control. Ultrathin sections of the bacteria on nickel grids were incubated with 4% sodium metaperiodate for 10 min, followed by 1% aqueous periodic acid for 10 min, and 1% fish skin gelatin (FSG) in PBS for 2 h. Sections were incubated with primary mouse anti-biotin Abs (clone 3D6.6) in 1% FSG overnight at 4°C followed by secondary antibodies (12 nm Colloidal Gold-Donkey anti-mouse Abs; both from Jackson ImmunoResearch) for 2 h at RT. Sections were fixed in 1% glutaraldehyde for 5 min, stained with uranyl acetate, and examined in TEM.

### Scanning Electron Microscopy (SEM)

Preparation of the bacteria samples was conducted in the same manner as for TEM procedure. In brief, bacteria in mid-logarithmic phase were incubated with p4 or PBS for 2 h at 37°C on coverslips in 12-wells plates. After incubation bacteria were fixed for 10 min in 2.5% glutaraldehyde in PBS, washed three times with PBS, and dehydrated in gradient ethanol series (15–100%). The samples were then transferred to a mixture (1:1, v:v) of ethanol and acetone, and pure acetone for two times 10 min each. Dry specimens were then coated with gold. Examination and photography were carried out with a HITACHI S-4700 scanning electron microscope.

### CRISPR/Cas9 Genome Editing

The plasmids, primers, and sgRNA target used to CRISPR/Cas-induced genetic modifications in *E. coli* are listed in Table 1. Gene targeting sequence was cloned into pgRNA-bacteria plasmid using Q5^®^ Site-Directed Mutagenesis Kit (New England BioLabs). For knockout mutation, electrocompetent *E. coli* HB101 was transformed with pCas plasmid and isolates were selected on LB agar plates containing kanamycin (50 μg/ml) (BioShop). Bacteria harboring pCas were then grown at 30°C in LB medium with kanamycin (50 μg/ml). Arabinose (10 mM final concentration) (Sigma-Aldrich) was added to the culture at an OD600 of 0.3 to 0.4 for induction of the recombinase (λ-Red) expression. When the optical density reached 0.5 to 0.7, cells were harvested, washed three times in ice-cold 10% glycerol, and mixed with 100 ng of pgRNA-bacteria plasmid and 1 μg of donor DNA (containing stop codon and sequence for *Hin*dIII digestion). Electroporation was done in a pre-chilled 2-mm electroporation cuvette (Sigma-Aldrich) at 2.5 kV and 5 ms. Cells were recovered in LB medium without antibiotics for 1 h at 30°C, plated onto LB agar containing kanamycin (50 μg/ml) and ampicillin (100 μg/ml) (Sigma-Aldrich) and incubated overnight at 30°C. Transformants were verified by colony PCR, *Hin*dIII (Thermo Fisher Scientific) digestion, and DNA sequencing (Genomed). Experiments with silencing *rpoS* expression were performed as follows. The chemical competent *E. coli* DH5α was transformed with plasmid pdCas9-bacteria expressing catalytically “dead” dCas9. The positive clones were selected on LB agar plates containing 25 μg/ml chloramphenicol (Sigma-Aldrich) and used for additional chemical transformation with pgRNA-bacteria plasmid. For the knockdown experiments, bacteria were grown in LB supplemented with antibiotics overnight at 37°C. The following day, bacteria were diluted and incubated in LB with appropriate antibiotics for additional 6 h to OD_600_ of 0.5–0.8. To obtain KD phenotype LB was additionally supplemented with 0.1 μM anhydrotetracycline (aTc) (Cayman Chemical).

**TABLE 1:** List of plasmids, primers, and sgRNA sequences used in the CRISPR/Cas9 experiments.

Plasmid	Purpose	Source
pgRNA-bacteria	Expression of guide RNA (gRNA)	Addgene #44251
pCas	Constitutive expression of cas9 and inducible expression of λ-Red	Addgene #62225
pdCas9-bacteria	aTc-inducible expression of a catalytically inactive bacterial Cas9 (dCas9)	Addgene #44249
**Primers**		
CCATAACGGGTTTTAGAGCTAGAAATAGC	Insertion of gene targeting sequence in pgRNA-bacteria plasmid	This study
CAATCGTGGTCACTAGTATTATACCTAGGAC	Insertion of gene targeting sequence in pgRNA-bacteria plasmid	This study
GGCGTATCACGAGGCAGAAT	Identification of modification of pgRNA-bacteria plasmid	This study
CGACTCGGTGCCACTTTTTC	Identification of modification of pgRNA-bacteria plasmid	This study
GTTGCGTATGTTGAGAAGCGG	Amplification of *rpoS*	This study
AACTGTTATCGCAGGGAGCC	Amplification of *rpoS*	This study
AACGCCAGCTAAAGC	Binding to modified fragment of *rpoS*	This study
AAATCGGCGGAACCA	Binding to modified fragment of *rpoS*	This study
**Target part of gene (+PAM sequence)**		
GACCACGATTGCCATAACGGCGG	Binding of dCas9	This study
**Donor DNA template**		
TCTTCGATAAGGTCCAGCAACGCCAGCTAAAGCTT GCCTTAACGGCGGGCAATTTTTACCACCAGACGCA	Modification of *rpoS*	This study
TGCGTCTGGTGGTAAAAATTGCCCGCCGTTAAGGC AAGCTTTAGCTGGCGTTGCTGGACCTTATCGAAGA	Modification of *rpoS*	This study

## Results

### Chem157S and p4 Display Differential Antimicrobial Activity Against Skin Bacteria

**FIGURE 1:**
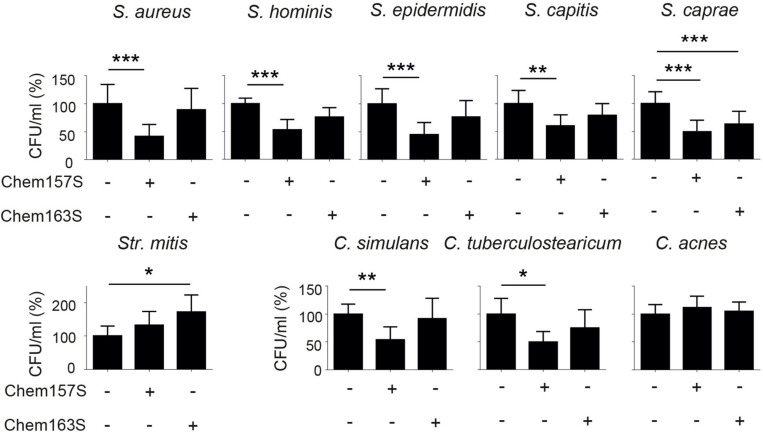
Chemerin controls growth of cutaneous bacteria. Bacteria were incubated with chemerin isoforms Chem157S, Chem163S (5 μM), or PBS (control) for 2 h. Cell viability in CFU/ml, shown as the percentage of control cells, was analyzed by MDA assay. Results are expressed as the mean ± SD of at least two independent experiments, performed with technical triplicates. ***p < 0.001, **p < 0.01, *p < 0.05 by one-way ANOVA with *post hoc*: Tukey’s multiple comparisons test.

Previous studies have identified specific alterations in the cutaneous microbiome in lesional psoriatic skin vs. healthy skin (Gao et al., 2008; Fahlen et al., 2012). Since chemerin is substantially reduced in psoriatic (lesional and non-lesional) epidermis vs. healthy epidermis, we hypothesized that psoriatic epidermis is preferentially inhabited by bacteria that are sensitive to chemerin antimicrobial activity. To test this hypothesis, we selected a diverse set of nine common clinical bacterial isolates altered in psoriatic skin for chemerin-mediated killing activity. These include *S. aureus, S. hominis, S. epidermidis, S. capitis, S caprae, Streptococcus mitis* (*Str. mitis*), *Corynebacterium simulans* (*C. simulans*), *C. tuberculostearicum*, and *Propionibacterium acnes* recently renamed *Cutibacterium acnes* (*C. acnes*) (Table 2). Chem157S (5 μM) restricted the growth of all *Staphylococcus* spp. and *C. simulans*, but it was ineffective against *Str. mitis* and *C. acnes*. In contrast, Chem163S had no antimicrobial activity against most tested strains with exception of *S. caprae* (Figure 1). Chemerin peptide p4 and Chem157S shared a similar pattern of activity against cutaneous microbiota (Figure 1 and Table 2), but the effects of p4 were overall more robust than Chem157S based on the complete inhibition of growth of sensitive bacteria at concentration ≥MIC (Table 2). Specifically, among the sensitive strains, p4 displayed best efficacy against *C. tuberculostearicum, S. caprae, S. capitis*, and *S. epidermidis* (MIC = 3.1–6.3 μM) (Table 2). P4 was ineffective against *C. acnes* and *Str. mitis* (Table 2). Therefore, antimicrobial chemerin derivatives exhibit selectivity against common and psoriasis-relevant cutaneous microbes.

**TABLE 2:** Antimicrobial activity of peptide p4 against skin bacteria.

Strain	MIC [μM]	Sensitive/Resistant [100 μM]	Strain-level skin changes, healthy individuals vs. donors with psoriasis	
*S. aureus* 8325-4	12.5–25	S	↑	Gao et al., 2008; Chang et al., 2018
*S. hominis* DSM 20328	12.5–25	S	↑	Gao et al., 2008
*S. epidermidis* DSM 20044	6.3	S	↑	Gao et al., 2008
*S. capitis* DSM 20326	3.1–6.3	S	↑	Gao et al., 2008
*S. caprae* DSM 20608	3.1	S	↓∼	Gao et al., 2008
*Str. mitis* DSM 12643	≥100	R	↓	Gao et al., 2008
*C. simulans* DSM 44392	6.3	S	↑	Gao et al., 2008
*C. tuberculostearicum* DSM 44922	3.1	S	↑	Gao et al., 2008
*C. acnes* DSM 16379	>100	R	↓	Gao et al., 2008; Chang et al., 2018

*Selected cutaneous bacteria previously shown to be qualitatively altered in psoriatic vs. healthy skin were incubated with chemerin p4. The MIC was defined as the lowest concentration of p4 showing no visible growth (100% of killing). Strains were defined as sensitive (S) or resistant (R) if they were or were not completely killed by 100 μM p4, respectively, in all experiments, *n* = 3.*

### P4 Suppresses Bacteria Growth by Directly Targeting Envelope-Dependent and Independent Pathways

**FIGURE 2:**
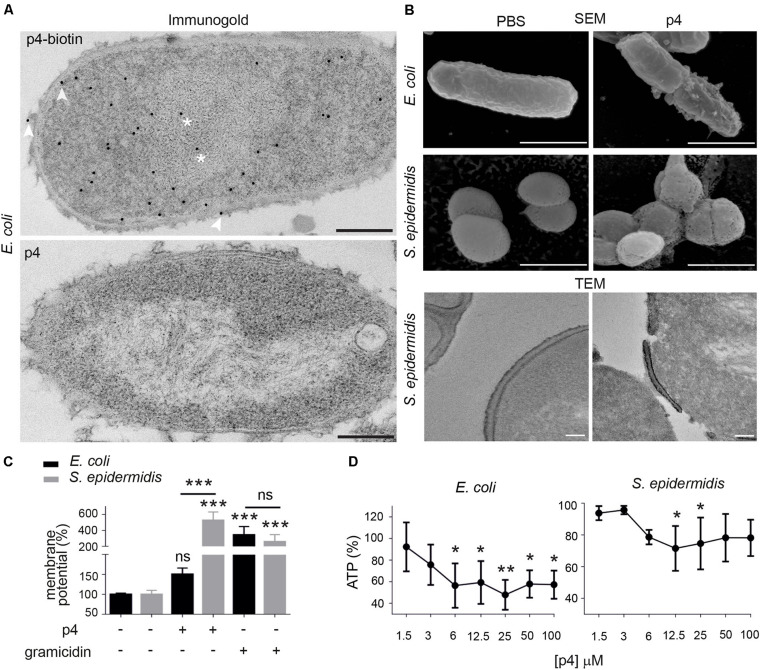
Chemerin peptide p4 localizes in different bacterial cell compartments and disrupts the cell envelope. **(A)** Localization of p4 is shown by immunogold labeling, followed by TEM. *E. coli* was incubated with 100 μM biotin-p4 (upper panel) or p4 as a control (lower panel) for 10 min, fixed, and stained with mouse anti-biotin Abs, followed by anti-mouse Abs conjugated to gold particles. Arrowheads and asterisks indicate interaction of p4 with cell envelope and nucleoid, respectively. **(B)** The indicated bacteria strains were incubated with 100 μM p4 or vehicle control (PBS) for 2 h, followed by SEM (upper and middle rows) or TEM (lower row). TEM and SEM images are from one experiment and are representative of at least three experiments. Scale bar = 300 nm (A), 1 μm (B SEM) or 100 nm (B,TEM). **(C)** *E. coli* and *S. epidermidis* were incubated with 25 μM p4, 1 μM gramicidin as a positive control, or vehicle control (PBS) for 10 min followed by measurement of membrane potential by fluorimetry. **(D)** *E. coli* and *S. epidermidis* were incubated with p4, or vehicle control (PBS) for 10 min followed by measurement of total ATP levels. Membrane potential **(C)** or ATP levels **(D)** are shown as the percentage of a vehicle-treated cells. Results are expressed as the mean ± SD of at least three independent experiments, performed in duplicates. ***p < 0.001, **p < 0.01, *p < 0.05, ns = non-significant by one-way ANOVA with *post hoc*: Tukey’s multiple comparisons test.

To better understand strain-dependent differences in p4 efficacy, we extended our analysis into mechanisms by which p4 controls bacteria growth. We previously showed that peptide p4 rapidly destabilized membrane integrity in p4-sensitive *E. coli* strains (MIC = 6.3–12.5 μM). However, we noted that p4 localized to multiple bacteria cell compartments 10 min after treatment, including the cell wall and nucleoid (Godlewska et al., 2019) and Figure 2A, suggesting that p4 affects bacteria viability by directly interfering with several key pathways. Combined scanning and transmission electron microscopy (SEM and TEM, respectively), demonstrated massive loss of cell surface structures and/or peeled cell envelope in *E. coli* and/or *S. epidermidis* (Figure 2B), indicating ultrastructural disruption of bacterial membranes and peptidoglycan layers in both Gram + and Gram- strains. Treatment of *E. coli* with p4 also resulted in a tendency for, and significant depolarization of, membrane potential in *E. coli* and *S. epidermidis*, respectively (Figure 2C), as well as accompanying loss of ATP levels (Figure 2D). Together, these data indicate that p4 affects bacteria growth by targeting bacteria shield components.

**FIGURE 3:**
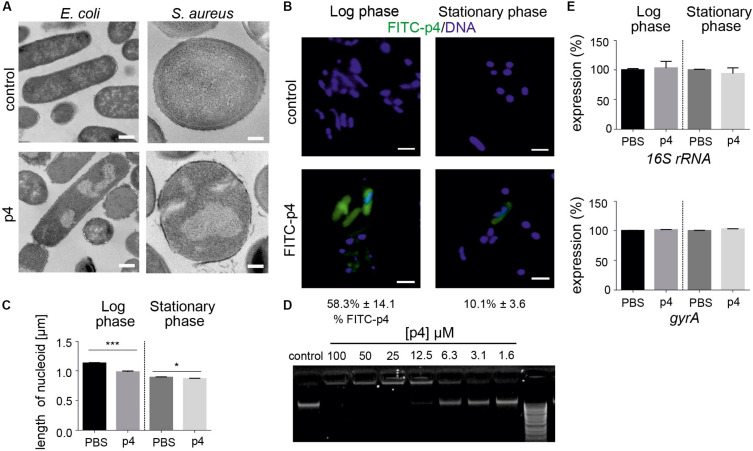
Bacteria treatment with p4 results in robust nucleoid condensation in logarithmic phase of growth. **(A)** The indicated bacteria strains were incubated with 100 μM p4 or vehicle control for 2 h, followed by TEM. **(B)** *E. coli* in the indicated phase of growth was incubated with 10 μM FITC-labeled p4 or vehicle control for 30 min, stained with Hoechst to visualize DNA, and analyzed by fluorescence microscopy. FITC-p4 positive cells are shown as the percentage of total cells (Mean ± SD). **(C)** Length of nucleoids were measured in p4 or vehicle-treated *E. coli* images using NIS-Elements Imaging Software, *n* = 3. In each experiment, at least 2 or 3 different high-power fields were analyzed. Results are expressed as the mean ± SD. ***p < 0.001, *p < 0.05 by Kruskal-Wallis one-way ANOVA with *Post hoc* Dunn’s test. **(D)** *E. coli*-derived DNA was incubated with the indicated concentration of p4 and analyzed by gel-retardation assay, *n* = 3. **(E)** Expression of the indicated house-keeping genes was analyzed by qPCR in *E. coli* grown in the logarithmic or stationary phase and treated with 5 μM p4 or vehicle (PBS) for 30 min, *n* = 3. Expression is shown as the percentage of vehicle-treated cells. Images in panel **(A)** are from one experiment and are representative of at least three experiments. Scale bar = 500 nm for *E. coli* and 200 nm for *S. aureus* **(A)** and 2 μm **(B)**.

P4 also induced condensation of bacteria chromosome in *E. coli* and Staphylococci (Figures 2A, 3A). Given the rapid localization of p4 on nucleoid (Figure 2A), these data suggested that p4 binding to DNA may interfere with the chromosome structure. In *E. coli*, nucleoid condensation occurs naturally during the stationary phase of growth. Fluorescence microscopy revealed that, in common with *E. coli* in the stationary phase, bacteria incubated in the presence of 10 μM p4 but not vehicle control for 30 min significantly reduced the nucleoid length in the log phase. This was visualized as a change of shape of nucleoids from rod-like, relaxed form in the log phase of growth to more compact, lobular forms in the stationary phase, and in the log phase in response to p4 (Figure 3B). In contrast, condensation of bacteria nucleoids, already robust in stationary phase without any treatment, was much less dependent on p4. The qualitative data were corroborated by quantitative length measurement of Hoechst-labeled bacterial chromosomes by fluorescence microscopy (Figure 3C). To explore the direct DNA-interacting ability of p4, we performed gel retardation analysis to assess the electrophoretic mobility of DNA. Increasing concentrations of p4 retarded the migration of bacterial DNA (Figure 3D). Maximal inhibition was noticed when DNA was exposed to p4 at concentrations above the MIC (12.5–100 μM); however, a lower concentration of p4 (that corresponds to 1 × MIC) was sufficient for partial retardation of DNA migration. The inhibitory effect was negligible for sublethal concentrations of p4 (Figure 3D). To determine whether the interaction between p4 and DNA can influence overall bacterial fitness, we determined transcription levels of two house-keeping genes: *16S rRNA* and *gyrA*. Expression of these genes in response to p4 was not significantly altered in either log or stationary phase of growth by qPCR (Figure 3E). These data suggest that p4 is not able to inhibit transcription of constitutively expressed genes, despite interacting with DNA and impacting nucleoid condensation.

### Bacteria Modulate Sensitivity to p4 by Influencing p4 Oxidation and Cell Surface Localization

**FIGURE 4:**
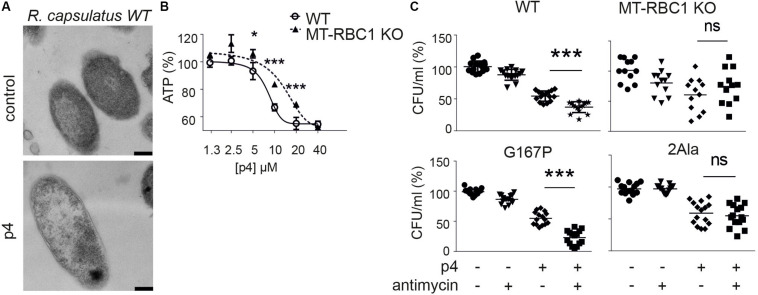
Bacteria capable of producing high level of ETC-dependent ROS are more sensitive to p4. **(A)** *R. capsulatus* was incubated with 100 μM p4 or vehicle (control) for 1 h followed by TEM. Scale bar = 200 nm. **(B)** *R. capsulats* (WT) or *R. capsulatus* deficient with cytochrome *bc*_1_ activity (MT-RBC1 KO) was incubated with the indicated concentration of p4 or vehicle control (PBS) for 30 min followed by measurement of total ATP levels. ATP levels are shown as the percentage of a vehicle-treated cells. Results are expressed as the mean ± SD of at least three independent experiments, performed in duplicates. ***p < 0.001, *p < 0.05 by one-way ANOVA with *post hoc*: Tukey’s multiple comparisons test. **(C)** The indicated strains of *R. capsulatus* were incubated with 2 μM of antimycin for 15 min followed by treatment with 2.5 μM of p4 or vehicle control for 2 h. *n* = 3. Results are expressed as the mean ± SD. ***p < 0.001, ns = non-significant by one-way ANOVA with *post hoc*: Tukey’s multiple comparisons test.

Since P4 is not uniformly bactericidal, it is possible that bacteria may employ countermeasures to ameliorate the damaging effects of p4. We showed that an oxidative environment can strongly augment p4 antimicrobial activity by supporting the formation of disulfide bonds leading to potent antimicrobial p4 dimers compared to barely active p4 monomers (Godlewska et al., 2019). To test whether bacteria themselves can modulate antimicrobial activity of p4 by oxidizing the peptide, we employed highly p4-sensitive Gram negative bacteria, *Rhodobacterium capsulatus* (*R. capsulatus*) (MIC = 5 μM) (Godlewska et al., 2019). In common with *E. coli* and *staphyloccoci*, p4 triggered robust morphological cell alterations, including cell layers distortion and condensation of nuclear material in *R. capsulatus* (Figure 4A). The p4-mediated effect on *R. capsulatus* also manifested in a rapid drop in ATP levels above MIC (Figure 4B). Changes in cytoplasmic ATP levels in response to p4 were at least partly dependent on electron transport chain (ETC), since *R. capsulatus* deficient in one of its key ETC enzymes, cytochrome *bc*_1_ (mutant MT-RBC1 KO), was less sensitive to sublethal and lethal p4 levels (Figure 4B). These data further support our previous findings, that p4 restricts the growth of *R. capsulatus* in association with cytochrome *bc*_1_ activity, which has an ability to facilitate the formation of antimicrobial p4 dimers via p4 oxidation (Godlewska et al., 2019).

To mimic the conditions under which ETC may enhance p4 activity, we forced the *bc*_1_-dependent superoxide generation by treatment of bacteria with antimycin (Borek et al., 2008, 2015). We reasoned that the sublethal antimycin administration in strains capable of producing reactive oxygen species (ROS) when treated with antimycin, would oxidize the peptide and boost p4 effect against *R. capsulatus*. Indeed, WT *R. capsulatus* and *R. capsulatus* G167P mutant with enhanced ability to produce superoxide in response to antimycin (Borek et al., 2015) were significantly more sensitive to p4 in the presence of antimycin. In contrast, there was no antimycin-dependent difference in antimicrobial p4 activity in two mutated *R. capsulatus* strains, MT-RBC1 KO and 2Ala (Borek et al., 2008, 2015), with markedly diminished ability to produce antimycin-dependent ROS (Figure 4C). Together, these data suggest that bacteria have the endogenous ability to calibrate the activation status of p4, for example, by modulating their oxidative potential by ROS production at the cell membrane in the vicinity of p4.

**FIGURE 5:**
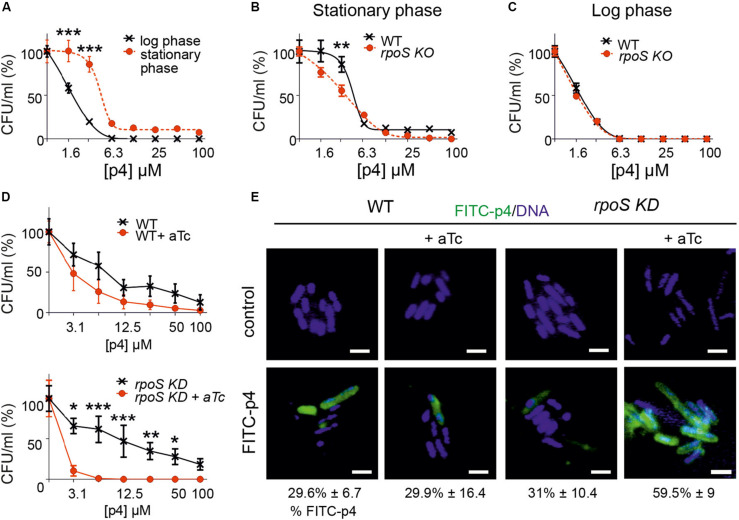
Bacteria resistance to p4 depends on the phase of growth and involves RpoS-mediated control of p4 interaction with the cell surface. **(A)** *E. coli* in the logarithmic or stationary phase of growth was treated with the indicated concentration of p4, or vehicle control for 2 h. Cell viability in CFU/ml, shown as the percentage of control cells, was analyzed by MDA assay. *n* = 3, mean ± SD. **(B)** *E. coli* (WT) or *E. coli* deficient with *rpoS* gene (*rpoS* KO) was incubated in the stationary phase of growth with the indicated concentration of p4 or vehicle control for 2 h followed by MDA assay. *n* = 3, mean ± SD. **(C)** WT and *rpoS* KO *E. coli* strains were incubated in the logarithmic phase of growth with the indicated concentration of p4 or vehicle control for 2 h followed by MDA assay. *n* = 3, mean ± SD. **(D)** WT *E. coli* (upper panel) or *E. coli* with *rpoS* gene controlled by aTc (*rpoS* KD) (lower panel) were treated with 0.1 μM aTC for 6 h to silence *rpoS* expression. The bacteria were then treated with the indicated concentration of p4 followed by MDA assay. *n* = 3, mean ± SD. **(E)** The indicated *E. coli* strains were treated with 0.1 μM aTc for 6 h followed by incubation with 20 μM p4 or vehicle control for 10 min. The bacteria were stained with Hoechst to visualize DNA and analyzed by fluorescence microscopy. Scale bar = 2 μm. FITC-p4 positive cells are shown as the percentage of total cells (Mean ± SD). ***p < 0.001, **p < 0.01, *p < 0.05 by one-way ANOVA with *post hoc*: Tukey’s multiple comparisons test.

Bacteria entering the stationary phase of growth modify their morphology, metabolic and transcriptional profile for protection against the harsh environment. These modifications include increasing resistance to antimicrobial factors (Jaishankar and Srivastava, 2017; Agrawal et al., 2019). Our observation that bacteria exposed to p4 in the logarithmic phase of growth condense their chromosomes in a similar way as untreated bacteria in the stationary phase (Figure 3) prompted us to ask whether p4 might induce the development of bacterial resistance conferred by the “stationary phase phenotype.” The susceptibility of *E. coli* to p4 in the logarithmic phase of growth was significantly higher than in cells in the stationary phase (Figure 5A). The most notable difference between *E. coli* treated with p4 in the log and stationary phase was observed at sublethal p4 concentrations (1.6 and 3.1 μM). However, even 100 μM p4 (the highest tested level), in spite of its strong bactericidal effect, did not completely kill *E. coli* in the stationary phase, since 7.4 ± 1.2% (mean ± SD) survived the p4 treatment (Figure 5A).

One of the main factors involved in protection of bacteria during stress conditions is transcriptional factor σ^*S*^ (RpoS) (Jaishankar and Srivastava, 2017). We next examined whether RpoS played a role in adaptability of bacteria to the p4-mediated threat. We used CRISPR/Cas to generate *E. coli* genetically deficient in RpoS (*rpoS* KO). During stationary phase, RpoSKO were significantly more susceptible to p4 than WT *E. coli* (Figure 5B). In contrast, *rpoS* KO bacteria maintained the p4 sensitivity of the WT strain in the logarithmic phase of growth (Figure 5C), in agreement with RpoS function confined to the stationary phase. Similar results were obtained using an inducible system for silencing *rpoS* gene (*rpoS* knockdown, KD). A decreased tolerance to antimicrobial effect of p4 was correlated with the activity of *rpoS* gene (controlled by aTc) in *rpoS* knockdown *E. coli* stain but not in the WT strain (Figure 5D).

*Escherichia coli* in the stationary phase bound less FITC-labeled p4 than *E. coli* in the logarithmic phase (Figure 3B). These data suggested that RpoS renders bacteria less susceptible to p4 by promoting the spatial segregation of the peptide and bacteria at the cell surface. To test this possibility, we examined binding of p4 to *E. coli* with the silenced *rpoS*. Compared to the WT *E. coli*, the *rpoS* knockdown mutant displayed markedly higher potential for binding of FITC-labeled p4 to the cell surface (Figure 5E). We conclude that the phenotypic resistance to p4 involves RpoS-dependent limiting of p4 association with the bacterial surface.

## Discussion

Chronic inflammatory skin diseases, including psoriasis, are associated with microbial cutaneous community changes that can negatively impact skin health. Although no single microbial biomarker indicative of psoriasis has been found, several microorganisms were linked to disease exacerbation, including chemerin-sensitive *S. aureus* (Tomi et al., 2005).

The skin defense system engages many components, including microbiota- and epidermis-derived antimicrobial factors (Kwiecien et al., 2019), suggesting that expression-level variations of these factors might play a pathogenic role in altering the cutaneous microbiome. However, in contrast to antimicrobial factors of microbial origin that can be expected to mainly affect the growth of other strains that compete for the same cutaneous niche, AMPs that are highly expressed in the epidermis such as chemerin may play a dominant role in restricting skin associated microbiota. Here we demonstrate the selectivity of antimicrobial chemerin derivatives against psoriasis-relevant cutaneous bacteria strains. Among nine tested strains, seven followed the predicted pattern of sensitivity to chemerin peptides in correlation with a diverse skin distribution in healthy (chemerin rich) and psoriasis (chemerin poor) epidermis. Among the sensitive chemerin strains were *C. tuberculostearicum, S. capitis, C. simulans, S. epidermidis, S. hominis*, and *S. aureus.* These strains were also reported to be present in higher quantities in non-lesional skin of psoriasis patients compared to healthy skin (Gao et al., 2008; Table 2), when epidermal chemerin levels may start to decline. In contrast, Chem157S and p4 resistant-*Str. mitis*, which could be expected to be largely spared by chemerin peptides in epidermis, was on average unchanged or less abundant in skin of psoriasis individuals (Gao et al., 2008; Table 2). However, association with chemerin levels was not observed for chemerin-resistant *C. acnes*, which was markedly underrepresented in psoriasis skin, as well as chemerin-sensitive *S. caprae*, which remained unchanged in psoriatic vs. healthy skin (Gao et al., 2008; Chang et al., 2018). The major determinants of skin inhabitance with these bacteria likely involve other factors. For example, *C. acnes* colonizes lipid-rich sebaceous skin areas. Since psoriatic skin is rather desiccated, skin dryness may be key factor in limiting *C. acnes* abundance in psoriatic skin. Chemerin exhibits antimicrobial potential against pathogenic microbes, such as *S. aureus*, as well as more benign skin strains such as *S. epidermidis*. Together these data suggest that chemerin does not selectively spare less harmful bacteria, but rather acts to restrict overgrowth of a variety of prevalent cutaneous genera, species, and strains. In the absence of the protective role of chemerin as an antimicrobial factor in epidermis, chemerin-controlled bacterial species might have a growth advantage in the skin undergoing pathological alterations, and thereby contribute to disease exacerbation.

The prophylactic potential of p4 against skin infection with a variety of microorganisms, including MRSA infection, and the therapeutic efficacy of p4 in treating experimental *S. aureus* skin infections [(Godlewska et al., 2019) and Table 2] could form the basis of potential new drugs for the prevention/treatment of skin infections, independent of the endogenous abundance of p4 *in vivo*. Here, we show that p4 is likely to target multiple bacterial components, including the cell wall, cell membrane, and chromosome. Although targeting several independent components can be expected to limit the ability of bacteria to develop resistance to p4, our data suggests that bacteria can engage two discrete mechanisms to protect against p4 lethality. First, ETC-dependent ROS formation augments antimicrobial potency of p4 (Figure 4). Therefore, bacteria restricted in their ability to produce ROS at the cell membrane may, at least to some degree, be protected against the comparatively more lethal p4 dimers. Second, blocking accessibility of p4 to the bacterial surface and limiting p4 interactions with the cell membrane and intracellular targets, such as DNA, might account for better bacterial tolerance to p4. In accordance with these proposed mechanisms, bacteria in the stationary phase of growth that become more resistant to p4 are known to: (i) cope with oxidative stress, for example, by encoding enzymes that remove ROS, and (ii) maintain membrane integrity, for example, by introducing changes to membrane lipid composition (Mitchell et al., 2017; Guo et al., 2019). Protective mechanisms available in the stationary phase of growth often activate RpoS-dependent pathways that both strengthen cell wall permeability barrier and activate efflux pumps (Kobayashi et al., 2006; Guo et al., 2019). These findings provide corroborating support for the possible RpoS-mediated bacterial defensive strategy in response to the p4 threat. Given that *rpoS*-silenced *E. coli* diminish p4 integration to the cell surface layers (Figure 5), bacteria may benefit by modifying their membrane properties in such a way to prevent p4 cell surface binding. Alternatively, resistance to p4 may involve alterations of p4 activity in the bacteria microenvironment, before or concurrent with p4 interactions with bacteria wall components. This could be a consequence of a release of ions and other modulating factors to the bacteria environment by RpoS-dependent efflux pumps.

We have thus characterized differential bactericidal activities of endogenous active chemerin (Chem157S) and p4 peptide against common types of cutaneous bacteria known to be altered in psoriasis. We have also discovered bacteria-mediated mechanisms that regulate the antimicrobial activity of chemerin. Given the potential clinical utility of chemerin and p4 in treating skin infections, insight into how skin bacteria can counteract p4 may prove useful in designing chemerin-based biologics geared to establish or restore a favorable cutaneous microbiome.

## Data Availability Statement

The raw data supporting the conclusions of this article will be made available by the authors, without undue reservation.

## Author Contributions

UG, BB, EP, and JC conceived and designed the experiments. UG and BB performed the experiments. PM contributed to the reagents and materials. JC and BZ wrote the manuscript. All authors contributed to the article and approved the submitted version.

## Conflict of Interest

The authors declare that the research was conducted in the absence of any commercial or financial relationships that could be construed as a potential conflict of interest.
